# Using the reflux symptom index to quantify throat symptom burden in LPR-screened-negative patients before and during SARS-CoV-2 infection in China: a prospective follow-up study

**DOI:** 10.3389/fmed.2026.1923973

**Published:** 2026-08-25

**Authors:** Ying Wang, Xingjia Hu

**Affiliations:** Department of Otolaryngology Head and Neck Surgery, Changde Hospital, Xiangya School of Medicine, Central South University (The First People’s Hospital of Changde City), Changde, China

**Keywords:** COVID-19, SARS-CoV-2, Omicron variant, reflux symptom index, laryngopharyngeal symptoms, throat symptom, associated factors

## Abstract

**Objectives:**

This study aimed to quantify throat symptom burden using the Reflux Symptom Index (RSI) in laryngopharyngeal reflux (LPR)-screened-negative patients before and during SARS-CoV-2 infection in the Omicron-dominant period, and to identify factors independently associated with RSI score increase during infection.

**Methods:**

This prospective follow-up study enrolled 428 LPR-screened-negative participants with SARS-CoV-2 infection during the Omicron-dominant period (December 2022–March 2023) in China. RSI questionnaires were administered at two time points: pre-infection baseline and follow-up. The second questionnaire was completed ≥4 weeks after infection onset, and participants retrospectively recalled acute symptoms experienced during the first 2 weeks of illness. Paired-samples *t*-tests, McNemar’s test, and multivariable linear regression were used for analysis.

**Results:**

Mean age was 37.59 ± 11.56 years; 64.7% were female. Mean total RSI score increased significantly from 5.04 ± 5.98 at baseline to 9.68 ± 7.48 during infection (mean ΔRSI = +4.64; *p* < 0.001). The proportion with RSI > 13 rose from 9.1 to 25.9% (*p* < 0.001). The most common symptoms during infection were troublesome cough (79.2%), throat clearing (76.9%), and excess throat mucus (72.4%). Multivariable linear regression identified three factors independently associated with greater RSI increase: stuffy/runny nose (*β* = 3.30, 95% CI 1.99–4.60, *p* < 0.001), fatigue (*β* = 2.53, 95% CI 0.83–4.23, *p* = 0.004), and muscle pains (*β* = 1.96, 95% CI 0.59–3.32, *p* = 0.005).

**Conclusion:**

SARS-CoV-2 infection during the Omicron-dominant period significantly worsened throat symptoms in LPR-screened-negative patients. Patients presenting with concurrent fatigue, nasal congestion, and muscle pain showed higher RSI increases and may warrant closer clinical attention.

## 1 Introduction

In early 2023, the SARS-CoV-2 Omicron variant had become the predominant circulating strain globally (1). Following revision of China’s epidemic prevention policy in December 2022 (2), a marked surge in SARS-CoV-2 infections during the Omicron-dominant period was observed, with otolaryngology clinics recorded a substantial increase in throat-related complaints. Chinese media coined the term “blade throat” to describe the severe, blade-like throat pain experienced during this period. Multiple published studies have characterized the symptom profile of SARS-CoV-2 infection across variants. Menni et al. reported that sore throat and hoarseness were among the most frequently reported symptoms during the Omicron wave, distinguishing it from earlier variants (3). A study by Yang et al. involving 310 patients showed that, after propensity score matching, the Omicron variant was associated with a significantly higher incidence of sore throat than the Beta variant (45.88% vs. 8.82%, *p* < 0.0001) (4). Furthermore, Abdel et al. retrospectively reported a surge in ulcerative laryngitis during the spring 2022 Omicron wave; typically secondary to respiratory infections with severe cough, it was characterized by dysphonia, vocal cord ulcerations, and a protracted clinical course (5). These findings underscore the need for structured, validated tools to quantify laryngopharyngeal symptom burden in the context of respiratory viral infections.

The Reflux Symptom Index (RSI) is a nine-item validated self-report questionnaire originally designed for laryngopharyngeal reflux (LPR) diagnosis (6). Each item is scored on a 0–5 Likert scale (0 = no problem, 5 = severe problem), with a total score range of 0–45; an RSI > 13 is the accepted threshold for suspected LPR (7). The nine items assess: (1) hoarseness or voice problems; (2) throat clearing; (3) excess throat mucus or postnasal drip; (4) difficulty swallowing food, liquids, or pills; (5) coughing after eating or lying down; (6) breathing difficulties or choking episodes; (7) troublesome or annoying cough; (8) sensation of something sticking in the throat or a lump in the throat (globus); and (9) heartburn, chest pain, indigestion, or stomach acid coming up. The symptom domains it captures—globus sensation, throat clearing, hoarseness, sore throat, and chronic cough—closely parallel the throat complaints observed in our clinic during the pandemic. Because eight of the nine RSI items directly pertain to pharyngeal and laryngeal symptomatology, it constitutes a structured, clinically validated instrument for quantifying throat symptom burden in context of viral respiratory infection. We emphasize that we employed the RSI as a structured symptom-burden tool, not as a validated COVID-19 outcome measure; RSI > 13 in this study reflects elevated throat symptom burden rather than a diagnosis of LPR or COVID-19-related disease.

To our knowledge, the RSI has not previously been applied to systematically characterize throat symptoms in COVID-19 patients who were screened negative for LPR. This study aimed to: (1) compare RSI scores before and during SARS-CoV-2 infection in the Omicron-dominant period among LPR-screened-negative patients; and (2) identify factors independently associated with an increase in RSI scores during infection.

## 2 Methods

### 2.1 Study design and setting

This was a prospective follow-up study conducted across two sequential time periods: a pre-infection baseline period (October–November 2022, prior to the Omicron outbreak) and an intra-pandemic follow-up period (SARS-CoV-2 Omicron-dominant surge, December 2022–March 2023). The median interval between baseline and follow-up assessments was 3 months (IQR: 2–4). Prior to the COVID-19 outbreak, we were conducting a population-based survey to estimate the prevalence of LPR among outpatient clinic attendees at the First People’s Hospital of Changde City. RSI questionnaires were administered remotely via the WeChat platform, and laryngoscopic examination with Reflux Finding Score (RFS) assessment was performed in the clinic. Following the revision of China’s epidemic prevention and control policy in December 2022 and the subsequent Omicron outbreak, we prospectively re-contacted all eligible LPR-screened-negative participants who reported confirmed SARS-CoV-2 infection during the Omicron-dominant period to complete a second RSI questionnaire. All follow-up assessments were completed via WeChat and telephone. This study was approved by the Ethics Committee of the First People’s Hospital of Changde City (YX-2023-012-01), and all adult participants provided written informed consent prior to enrolment; minor participants (aged 12–17 years) provided written assent with concurrent written parental/guardian consent.

### 2.2 Participants

#### 2.2.1 Baseline screening

All participants completed the RSI and underwent laryngoscopy for RFS assessment at baseline. Participants with RSI > 13 and/or RFS > 7 underwent 24-h dual-probe pH monitoring to confirm or exclude LPR; those with pH-confirmed LPR were excluded. Participants with RSI ≤ 13 and RFS ≤ 7 were classified as screened negative for LPR using validated RSI/RFS criteria, with pH monitoring performed in selected suspected cases. The RSI uses a 0–5 Likert scale per item (total: 0–45); RSI > 13 is the accepted LPR threshold (6). The RFS (range: 0–26) assesses eight laryngoscopic findings (subglottic oedema, ventricular obliteration, erythema/hyperaemia, vocal fold oedema, diffuse laryngeal oedema, posterior commissure hypertrophy, granuloma/granulation tissue, and thick endolaryngeal mucus); RFS > 7 indicates suspected LPR (8). For 24-h pH monitoring, dual antimony electrodes connected to a Digitrapper MK3 recorder (Medtronic, USA) were used. LPR was confirmed if pharyngeal reflux episodes were ≥6.9/24 h or Reflux Area Index ≥6.3.

#### 2.2.2 Baseline participant flow

**Figure 1:**
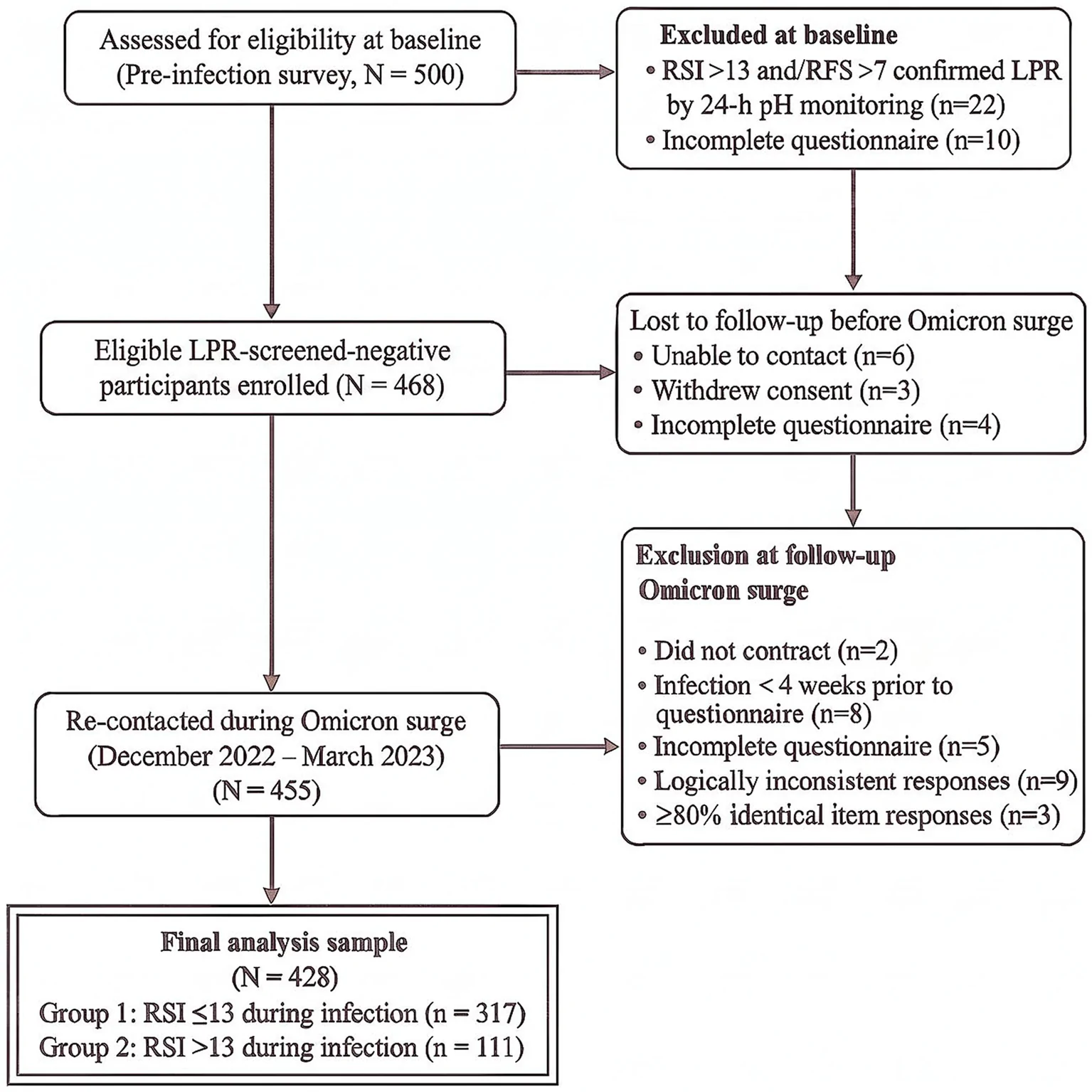
Flowchart of the study.

Of the 500 participants assessed at baseline, 22 were excluded for pH-confirmed LPR and 10 for incomplete questionnaires, leaving 468 eligible LPR-screened-negative participants enrolled. During follow-up re-contact (December 2022–March 2023), 6 could not be reached, 3 withdrew consent, and 4 had incomplete questionnaires (*n* = 455 re-contacted). Of these, 27 were excluded: 2 did not contract SARS-CoV-2, 8 had infection onset <4 weeks before questionnaire, 5 had incomplete questionnaires, 9 had logically inconsistent responses, and 3 had ≥80% identical item responses; yielding a final analytic sample of 428 (see Figure 1 for the complete participant flow).

#### 2.2.3 Follow-up inclusion criteria

Eligible participants required: (1) confirmed SARS-CoV-2 infection by RT-PCR or rapid antigen testing during December 2022–March 2023 (the Omicron-dominant period in China; individual-level variant confirmation by sequencing was not performed); (2) ability to independently complete the RSI; and (3) infection onset ≥4 weeks prior to follow-up questionnaire completion.

#### 2.2.4 Follow-up RSI timing

At follow-up, participants completed the RSI by retrospectively recalling symptoms experienced during the acute phase of their SARS-CoV-2 infection (i.e., within the first 2 weeks of illness). Questionnaires were completed ≥4 weeks after infection onset; this interval was required to confirm infection resolution and enable stable retrospective recall of acute-phase symptoms, and does not indicate that persistent or long-COVID symptoms were captured.

#### 2.2.5 Exclusion criteria at follow-up

Incomplete questionnaire; logically inconsistent responses; ≥80% of items answered with identical scores.

#### 2.2.6 Participant grouping

For descriptive comparisons, participants were stratified by RSI during infection: Group 1 (RSI ≤ 13, *n* = 317) and Group 2 (RSI > 13, *n* = 111). It should be noted that RSI > 13 in this context reflects elevated throat symptom burden during SARS-CoV-2 infection, and does not constitute an LPR diagnosis.

### 2.3 Data collection

The follow-up questionnaire collected: sex; age; BMI; occupation; smoking and alcohol history; comorbidities; COVID-19 vaccination status; RSI scores at both time points; accompanying systemic symptoms (fever, fatigue, stuffy/runny nose, hyposmia, hypogeusia, sore throat, muscle pains, diarrhea); and symptom duration. Electronic laryngoscopy was performed in patients reporting severe “blade throat” pain.

### 2.4 Statistical analysis

Analyses used SPSS 22.0 and EmpowerStats. Normality of continuous variables was assessed using the Kolmogorov–Smirnov test; RSI scores and ΔRSI approximated a normal distribution and were analyzed using parametric methods. Normally distributed continuous data are presented as mean ± SD; categorical variables as *n* (%). Paired-samples *t*-tests compared RSI scores between time points; McNemar’s test compared proportions of RSI > 13. A Bonferroni-corrected threshold (*p* < 0.0056) was applied for nine simultaneous item-level comparisons. Between-group differences used independent-samples *t*-tests or chi-square tests as appropriate. The dependent variable for regression was ΔRSI (RSI during infection − RSI before infection). Variables with *p* < 0.10 in univariate analysis entered the multivariable linear regression model, adjusted for pre-infection RSI score. Collinearity was assessed using variance inflation factors (VIF); all VIF values were <5, indicating no problematic collinearity. Sore throat was excluded from the multivariable model due to conceptual and item-level overlap with RSI items 4 and 8 (potential collinearity). Categorical predictors included in the final model were coded as indicator (dummy) variables, with reference categories stated in Table 1. Because vaccination status comprised five mutually exclusive categories, each dose category (unvaccinated, one dose, two doses, three doses, four doses) was individually tested as a separate binary indicator (yes vs. no, with “no” as the reference) in independent univariate and multivariable models, rather than being entered simultaneously within a single model; this avoided perfect multicollinearity that would otherwise arise from entering all mutually exclusive categories together. Specifically, a base multivariable model was first fitted, comprising pre-infection RSI score and all other covariates shown in Table 1, but excluding any vaccination variable; each vaccination dose category was then added individually to this same base model as an additional binary indicator, yielding five separate expanded models. All non-vaccination coefficients reported in Table 1, together with the adjusted R^2^ = 0.253, are derived from the base model (i.e., without any vaccination term); the adjusted R^2^ of the five expanded models ranged narrowly from 0.251 to 0.257 and did not materially differ from the base model. Because five separate binary comparisons were performed, these dose-category analyses are explicitly labeled as exploratory sensitivity analyses rather than a single confirmatory test, and the resulting *p* values were not adjusted for multiplicity in the primary analysis. To contextualize this, we additionally applied a Bonferroni correction for five simultaneous comparisons (adjusted significance threshold, *p* < 0.01); under this stricter threshold, the one-dose comparison (adjusted *p* = 0.015) would no longer be considered statistically significant. Only the one-dose comparison remained significant after adjustment at the conventional (uncorrected) threshold and is reported in Table 1 as an exploratory finding requiring cautious interpretation. Missing data at follow-up were handled by listwise deletion; the final analytic sample of 428 represented 94.1% of re-contacted participants (455), indicating minimal data loss. Regression model adequacy was verified as follows: linearity was assessed by inspection of partial regression (added-variable) plots; homoscedasticity was evaluated using plots of standardized residuals against fitted values, which revealed no systematic heteroscedastic pattern; normality of regression residuals was confirmed by visual inspection of normal P–P plots and a Kolmogorov–Smirnov test applied to standardized residuals (*p* > 0.05); and influential observations were identified using Cook’s distance, with all values < 1 indicating no unduly influential cases. Overall model fit is reported as adjusted R^2^ in the Results. Results are reported as unstandardized coefficients (*β*) with 95% CIs; two-sided *p* < 0.05 was considered significant.

**Table 1:** Univariate and multivariable linear regression analysis of predictors of RSI score change (ΔRSI) during SARS-CoV-2 infection (univariate analyses: *N* = 428; multivariable model: *N* = 417).

Variable	Category/value	Univariate		Multivariate	
		*β* (95% CI)	*p*-value	*β* (95% CI) ᶜ	*p*-value
Sex					
Male	151 (35.28%)	Ref.		Ref.	
Female	277 (64.72%)	1.96 (0.48, 3.43)	0.010	1.31 (−0.04, 2.67)	0.058
Age, years	37.59 ± 11.56	−0.03 (−0.09, 0.03)	0.291		
BMI, kg/m^2^	22.89 ± 3.76	0.05 (−0.13, 0.24)	0.576		
Occupation					
Healthcare worker	90 (21.03%)	Ref.			
Non-healthcare worker	338 (78.97%)	−0.09 (−1.83, 1.65)	0.920		
Smoking					
Yes	58 (13.55%)	Ref.			
No	370 (86.45%)	1.45 (−0.62, 3.52)	0.170		
Alcohol consumption					
Yes	43 (10.05%)	Ref.			
No	385 (89.95%)	0.94 (−1.42, 3.30)	0.434		
Passive smoking					
Yes	91 (21.26%)	Ref.			
No	337 (78.74%)	0.63 (−1.10, 2.37)	0.475		
Hypertension					
Yes	24 (5.61%)	Ref.			
No	404 (94.39%)	1.34 (−1.74, 4.43)	0.394		
Diabetes					
Yes	7 (1.64%)	Ref.			
No	421 (98.36%)	1.71 (−3.88, 7.31)	0.549		
Prior SARS-CoV-2 infection					
Yes	37 (8.64%)	Ref.			
No	391 (91.36%)	1.02 (−1.51, 3.54)	0.431		
RSI score before COVID-19	5.04 ± 5.98	0.36 (0.25, 0.47)	<0.001	0.33 (0.23, 0.44)	<0.001
Vaccination statusᵈ					
Four doses					
No	393 (91.82%)	Ref.			
Yes	35 (8.18%)	−0.15 (−2.74, 2.44)	0.907		
Three doses					
No	129 (30.94%)	Ref.			
Yes	296 (70.98%)	−1.27 (−2.80, 0.27)	0.106		
Two doses					
No	341 (81.77%)	Ref.			
Yes	76 (18.23%)	−0.04 (−1.88, 1.80)	0.968		
One dose					
No	418 (97.66%)	Ref.		Ref.	
Yes	10 (2.34%)	5.08 (0.62, 9.54)	0.026	4.96 (0.99, 8.92)	0.015
Unvaccinated					
No	417 (97.43%)	Ref.		Ref.	
Yes	11 (2.57%)	4.24 (−0.22, 8.71)	0.063	3.94 (−0.02, 7.90)	0.052
Accompanying symptoms					
Fever					
No	110 (25.7%)	Ref.		Ref.	
Yes	318 (74.3%)	2.11 (0.50, 3.72)	0.011	0.29 (−1.21, 1.79)	0.703
Fatigue					
No	84 (19.6%)	Ref.		Ref.	
Yes	344 (80.4%)	4.26 (2.52, 6.00)	<0.001	2.53 (0.83, 4.23)	0.004
Stuffy/runny nose					
No	198 (46.3%)	Ref.		Ref.	
Yes	230 (53.7%)	4.55 (3.20, 5.91)	<0.001	3.30 (1.99, 4.60)	<0.001
Hyposmia					
No	293 (68.5%)	Ref.		Ref.	
Yes	135 (31.5%)	3.39 (1.90, 4.89)	<0.001	1.39 (−0.02, 2.80)	0.054
Hypogeusia					
No	254 (59.3%)	Ref.		Ref.	
Yes	174 (40.7%)	2.08 (0.65, 3.51)	0.005	−1.55 (−3.22, 0.12)	0.070
Sore throatᵉ					
No	234 (54.7%)	Ref.			
Yes	194 (45.3%)	4.80 (3.40, 6.10)	<0.001		
Muscle pains					
No	175 (40.9%)	Ref.		Ref.	
Yes	253 (59.1%)	3.32 (1.91, 4.73)	<0.001	1.96 (0.59, 3.32)	0.005
Diarrhea					
No	374 (87.4%)	Ref.		Ref.	
Yes	54 (12.6%)	2.42 (0.29, 4.54)	0.026	1.66 (−0.23, 3.55)	0.087

RSI, Reflux Symptom Index; ΔRSI, change in total RSI score (during infection minus before infection); *β*, unstandardized linear regression coefficient; CI, confidence interval; BMI, body mass index; Ref., reference category.

ᵃThe dependent variable is ΔRSI (continuous). Multivariable linear regression was used; *β* represents the mean change in ΔRSI associated with each predictor, adjusted for all other variables in the model.

ᵇVariables with *p* < 0.10 in univariate analysis were entered into the multivariable model.

ᶜThe multivariable model was adjusted for pre-infection RSI score (as a continuous covariate) and the covariates listed with multivariable results in this table; All non-vaccination coefficients and the adjusted R^2^ = 0.253 are derived from the base model (excluding any vaccination variable); vaccination dose categories were tested separately (note “d”). See Methods 2.4 for details.

ᵈVaccination dose categories are mutually exclusive; each was tested individually as a binary indicator added to the base model (see Methods 2.4), rather than jointly, to avoid perfect multicollinearity. These are exploratory sensitivity analyses: the one-dose comparison did not remain significant after Bonferroni correction (*p* < 0.01) and, given the small subgroup (*n* = 10) and wide 95% CI (0.99–8.92), is hypothesis-generating only and does not indicate a dose–response relationship.

ᵉ“Sore throat” was excluded from the multivariable model due to its conceptual and item-level overlap with RSI items 4 (difficulty swallowing) and 8 (globus sensation), which would introduce collinearity. Its strong univariate association (*β* = 4.80, *p* < 0.001) is reported for completeness.

ᶠUnivariate analyses were conducted in the full analytic sample (*N* = 428). The multivariable model used complete-case analysis and included 417 participants after listwise deletion for missing covariate data.

## 3 Results

### 3.1 Demographic characteristics

A total of 428 LPR-screened-negative participants with confirmed SARS-CoV-2 infection were included. Participants were stratified by RSI score during infection: Group 1 comprised those with RSI ≤ 13 (i.e., below the LPR screening threshold; *n* = 317, 74.1%), and Group 2 comprised those with RSI > 13 (i.e., above the LPR screening threshold, indicating high throat symptom burden; *n* = 111, 25.9%). As noted, RSI > 13 here denotes elevated symptom burden, not an LPR diagnosis. Of these, 151 (35.3%) were male and 277 (64.7%) female, with mean age 37.59 ± 11.56 years (range: 12–88) and mean BMI 22.89 ± 3.76 kg/m^2^. Group 2 had a significantly higher proportion of female participants than Group 1 (74.8% vs. 61.2%; *p* = 0.010), with significant differences also in vaccination status (*p* = 0.044) and symptom duration (*p* < 0.001). In Group 1, the most common symptom duration was 5–7 days (24.6%); in Group 2, symptoms >15 days predominated (45.9%) (Table 2).

**Table 2:** Demographic and clinical characteristics of the study cohort stratified by RSI score during SARS-CoV-2 infection (*N* = 428).

Characteristic	Total (*N* = 428)	Group 1 (RSI ≤ 13 *N* = 317)	Group2 (RSI > 13 *N* = 111)	*p*-value
Age, years	37.59 ± 11.56	38.17 ± 11.30	35.94 ± 12.18	0.080^a^
BMI, kg/m^2^	22.89 ± 3.76	22.82 ± 3.50	23.08 ± 4.43	0.530^a^
Sex				0.010^b^
Male	151 (35.3%)	123 (38.80%)	28 (25.23%)	
Female	277 (64.7%)	194 (61.20%)	83 (74.77%)	
Occupation				0.717^b^
Healthcare worker	90 (21.0%)	68 (21.45%)	22 (19.82%)	
Non-healthcare worker	338 (79.0%)	249 (78.55%)	89 (80.18%)	
Vaccination status				0.044^b^
Unvaccinated	11 (2.6%)	6 (1.89%)	5 (4.50%)	
One dose	10 (2.3%)	4 (1.26%)	6 (5.41%)	
Two doses	76 (17.8%)	54 (17.03%)	22 (19.82%)	
Three doses	296 (69.2%)	225 (70.98%)	71 (63.96%)	
Four doses	35 (8.2%)	28 (8.83%)	7 (6.31%)	
Smoking				0.052^b^
Yes	58 (13.6%)	49 (15.46%)	9 (8.11%)	
No	370 (86.4%)	268 (84.54%)	102 (91.89%)	
Alcohol consumption				0.430^b^
Yes	43 (10.0%)	34 (10.73%)	9 (8.11%)	
No	385 (90.0%)	283 (89.27%)	102 (91.89%)	
Passive smoking				0.215^b^
Yes	91 (21.3%)	72 (22.71%)	19 (17.12%)	
No	337 (78.7%)	245 (77.29%)	92 (82.88%)	
Hypertension				0.286^b^
Yes	24 (5.6%)	20 (6.31%)	4 (3.60%)	
No	404 (94.4%)	297 (93.69%)	107 (96.40%)	
Diabetes				0.478^b^
Yes	7 (1.6%)	6 (1.89%)	1 (0.90%)	
No	421 (98.4%)	311 (98.11%)	110 (99.10%)	
Duration of RSI symptoms				<0.001^b^
1–3 days	70 (16.4%)	65 (20.50%)	5 (4.50%)	
3–5 days	62 (14.5%)	53 (16.72%)	9 (8.11%)	
5–7 days	99 (23.1%)	78 (24.61%)	21 (18.92%)	
7–15 days	78 (18.2%)	53 (16.72%)	25 (22.52%)	
>15 days	119 (27.8%)	68 (21.45%)	51 (45.95%)	

BMI, body mass index; RSI, Reflux Symptom Index.

Data are presented as mean ± standard deviation or *n* (%). Participant grouping is based on RSI scores assessed during SARS-CoV-2 infection (follow-up period), not at pre-infection baseline. Vaccination status was categorized as: Unvaccinated (0 doses), One dose, Two doses, Three doses (standard primary series plus one booster), and Four doses (primary series plus two boosters), based on China’s national COVID-19 vaccination program. Groups are not mutually exclusive to a reference category in this descriptive table; reference categories are specified in Table 1 for regression analyses.

^a^Independent-samples *t*-test.

^b^Pearson chi-square test; Fisher’s exact test applied when any expected cell count <5.

### 3.2 Distribution of RSI scores and symptom incidence before and during SARS-CoV-2 infection

Mean total RSI score increased significantly from 5.04 ± 5.98 to 9.68 ± 7.48 during the acute phase of infection (retrospectively recalled) (ΔRSI = +4.64; *p* < 0.001). RSI items 2–9 all increased significantly after Bonferroni correction (all *p* < 0.0056); item 1 (hoarseness) showed no significant change (*p* = 0.112). The proportion with RSI > 13 rose from 9.1% (39/428) to 25.9% (111/428) (*p* < 0.001, McNemar’s test) (Table 3).

**Table 3:** Comparison of individual RSI item scores and total RSI scores before and during SARS-CoV-2 infection (*N* = 428).

RSI Item	RSI score before COVID-19	RSI score during COVID-19	*p*-value
1. Hoarseness or a problem with your voice	0.73 ± 1.08	0.83 ± 1.11	0.112^a^
2. Clearing your throat	0.80 ± 1.05	1.44 ± 1.16	<0.001^a^*
3. Excess throat mucus or postnasal drip	0.76 ± 1.10	1.40 ± 1.24	<0.001^a^*
4. Difficulty swallowing food, liquids or pills	0.28 ± 0.74	1.63 ± 1.07	<0.001^a^*
5. Coughing after you ate or lying down	0.61 ± 1.02	1.27 ± 1.27	<0.001^a^*
6. Breathing difficulties or choking episodes	0.26 ± 0.63	0.65 ± 0.98	<0.001^a^*
7. Troublesome or annoying cough	0.66 ± 1.10	1.73 ± 1.36	<0.001^a^*
8. Sensation of something sticking in your throat or a lump in your throat	0.60 ± 0.96	1.22 ± 1.22	<0.001^a^*
9. Heartburn, chest pain, indigestion, or stomach acid coming up	0.35 ± 0.76	0.51 ± 0.88	<0.001^a^*
Total RSI score	5.04 ± 5.98	9.68 ± 7.48	<0.001^a^
Patients with RSI > 13, *n* (%)	39 (9.1%)	111 (25.9%)	<0.001^b^

RSI, Reflux Symptom Index.

^a^Paired-samples *t*-test.

^b^*p*-value derived from McNemar’s test for comparison of proportions.

*Statistically significant after Bonferroni correction for 9 simultaneous item-level comparisons (corrected α = 0.05/9 = 0.0056). Item 1 (hoarseness) did not reach significance at either the uncorrected or Bonferroni-corrected threshold (*p* = 0.112). Each RSI item is rated on a 0–5 scale (0 = no problem, 5 = severe problem); total RSI score ranges from 0 to 45. RSI > 13 indicates elevated throat symptom burden; in this study, it does not constitute a diagnosis of LPR.

**Figure 2:**
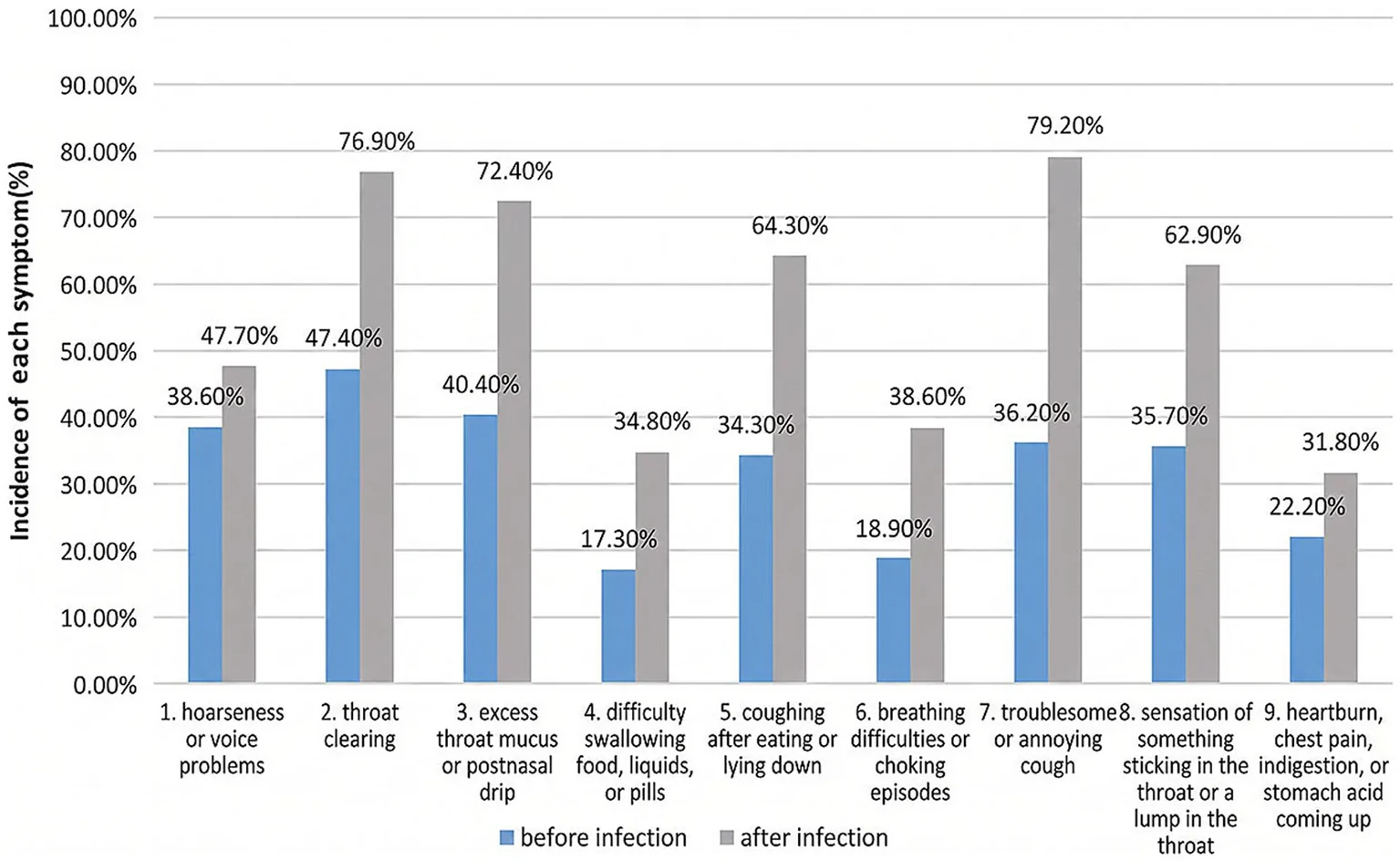
The incidence of each RSI at pre-infection baseline and during the acute phase of SARS-CoV-2 infection (retrospectively recalled at follow-up).

**Figure 3:**
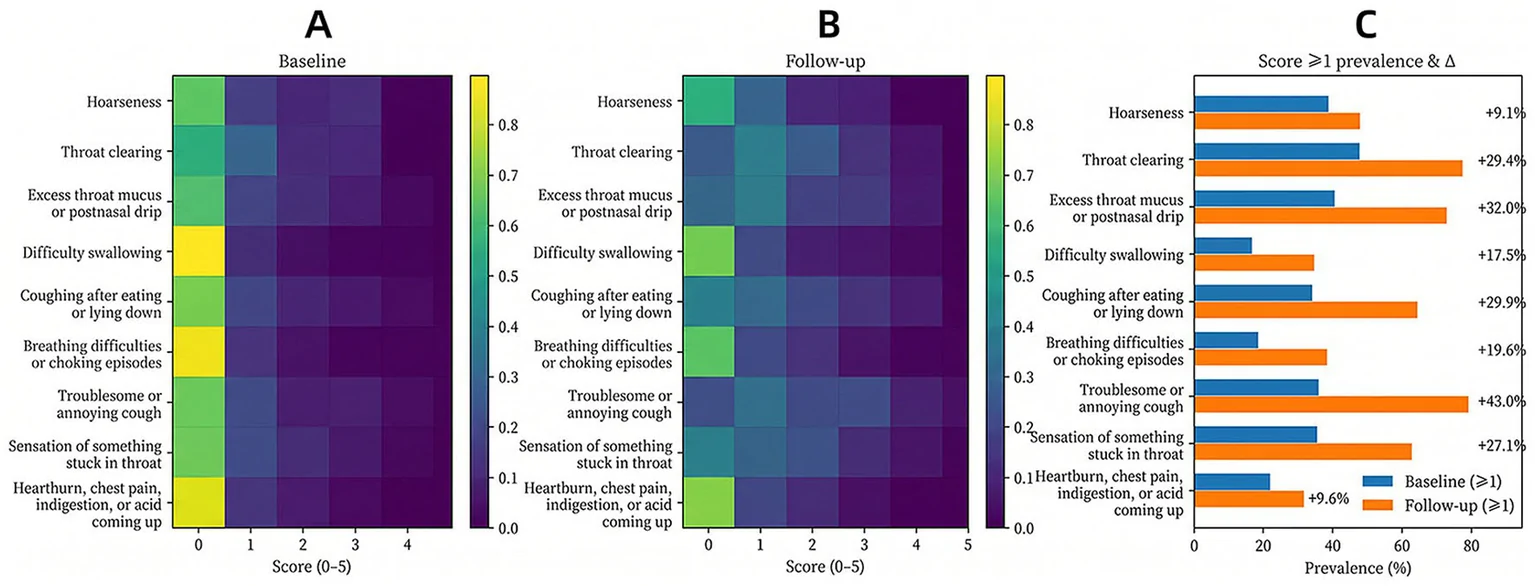
Heatmap showing the proportion of participants (%) endorsing each severity score (0–5) for all nine Reflux Symptom Index (RSI) items at **(A)** pre-infection baseline (October–November 2022) and **(B)** acute phase of SARS-CoV-2 infection during the Omicron-dominant period (retrospectively recalled at follow-up, December 2022–March 2023). Color intensity reflects the proportion of participants at each score level, ranging from white (0%) to dark blue (higher proportions). **(C)** Bar chart depicting the prevalence of each RSI item (score ≥ 1) at baseline (blue) and follow-up (red), with the absolute change (*Δ*%) annotated for each item. Data are based on 428 LPR-screened-negative participants.

Before infection, the most prevalent symptoms were throat clearing (47.4%), excess throat mucus (40.4%), and hoarseness (38.6%). During the acute phase of infection (retrospectively recalled), the predominant symptoms shifted to troublesome cough (79.2%), throat clearing (76.9%), and excess throat mucus (72.4%). Figure 2 shows the incidence of each RSI symptom before and during infection. A rating of ≥1 (any problem) on a given item was used to define symptom presence. For a detailed view of symptom severity distribution across all score levels (0–5) for each RSI item before and during infection, see the heatmap (Figure 3).

**Figure 4:**
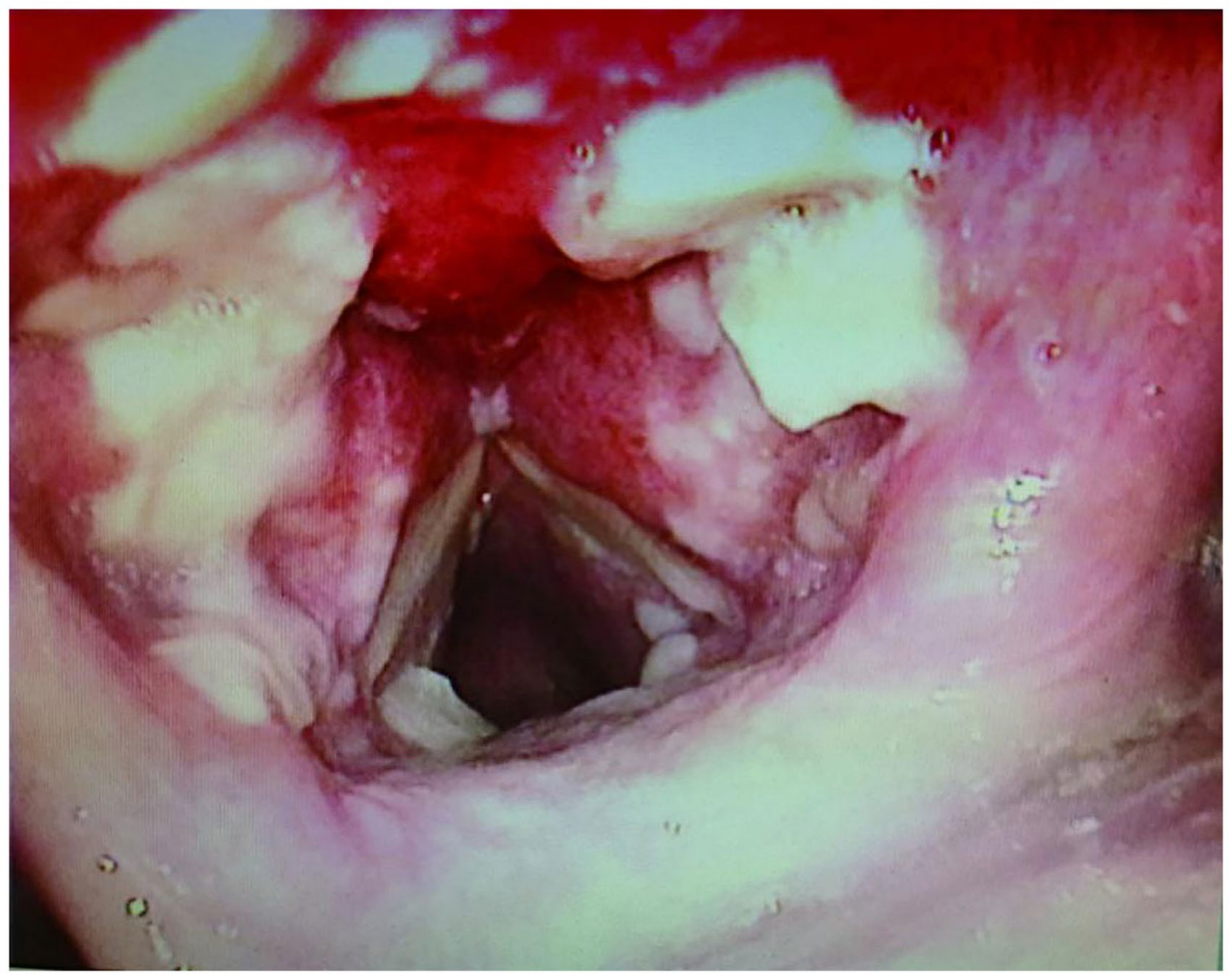
Representative electronic laryngoscopy image showing erosive mucosal changes in the glottis and supraglottic area, observed in a patient presenting with severe pharyngeal pain (‘blade throat’) during SARS-CoV-2 infection. Laryngoscopy was performed at the time of clinical presentation (during the acute/subacute phase of infection). Similar findings were observed in all 18 patients who underwent laryngoscopy; this image is representative. These changes may reflect severe inflammatory injury; however, direct viral causation or Omicron specificity cannot be established from these observations alone.

Electronic laryngoscopy in 18 patients with severe “blade throat” pain revealed marked erythema and erosive changes of the glottic and supraglottic mucosa in all cases (Figure 4). These findings are of interest but should be interpreted cautiously: these patients were selected from a symptomatic subgroup, no control group was available, and no histological confirmation or lesion-level viral testing was performed. These appearances may reflect severe inflammatory response rather than direct viral cytopathic injury, and their specificity to Omicron cannot be established from the current data. All were managed conservatively with analgesics, mucosal protectants, and close follow-up.

### 3.3 Regression analyses

Univariate regression identified the following significant factors associated with greater ΔRSI at *p* < 0.10 (entry threshold for multivariable model): pre-infection RSI score, one-dose vaccination, female sex, fever, fatigue, stuffy/runny nose, hyposmia, hypogeusia, sore throat, muscle pains, and diarrhea (Table 1).

The base multivariable model — comprising pre-infection RSI score and all covariates listed in Table 1, excluding vaccination — was fitted on 417 participants with complete data (adjusted R^2^ = 0.253); all non-vaccination coefficients reported below and in Table 1 are derived from this base model. Multivariable linear regression identified stuffy/runny nose (*β* = 3.30, 95% CI 1.99–4.60, *p* < 0.001), fatigue (*β* = 2.53, 95% CI 0.83–4.23, *p* = 0.004), and muscle pains (*β* = 1.96, 95% CI 0.59–3.32, *p* = 0.005) as independent predictors of ΔRSI. Vaccination status was not included in the base model. Instead, each of the five mutually exclusive dose categories was added individually to the same base model as a separate binary indicator, yielding five exploratory sensitivity analyses (see Methods and Table 1, noted). Only the one-dose category reached nominal significance (adjusted *β* = 4.96, 95% CI 0.99–8.92, *p* = 0.015; base model plus the one-dose indicator, adjusted R^2^ = 0.257). This vaccination-related finding is exploratory: the one-dose subgroup was very small (*n* = 10), vaccination timing was not recorded, and residual confounding cannot be excluded. The association did not survive a Bonferroni-corrected significance threshold for five comparisons (*p* < 0.01) and should be regarded as hypothesis-generating only. No inference regarding vaccine-related harm or protection should be drawn. The wide 95% CI for one-dose vaccination reflects the small subgroup size (*n* = 10) and warrants cautious interpretation. Pre-infection RSI score remained a significant predictor (*β* = 0.33, 95% CI 0.23–0.44, *p* < 0.001) (Table 1). These factors are concurrent symptoms or characteristics of the same illness episode as the outcome (ΔRSI) and should therefore be interpreted as associated factors reflecting illness severity rather than as causal predictors independent of disease severity.

## 4 Discussion

This prospective follow-up study demonstrated that SARS-CoV-2 infection during the Omicron-dominant period significantly increased pharyngolaryngeal symptom burden, as measured by the RSI, in patients who had been screened negative for LPR at baseline. The mean RSI score nearly doubled from 5.04 to 9.68, and the proportion of participants exceeding the RSI > 13 threshold rose nearly threefold (9.1 to 25.9%), underscoring the substantial throat symptom burden associated with this infection.

Our finding of troublesome cough (79.2%), throat clearing (76.9%), and excess mucus (72.4%) as the dominant symptoms aligns with published data from the Omicron wave. Menni et al. reported sore throat in approximately 53% and hoarseness in 35% of UK Omicron cases, with a higher prevalence of upper respiratory symptoms than prior variants (3). Our higher rates likely reflect the selected nature of our outpatient otolaryngology-adjacent cohort, who were inherently more attuned to throat symptoms. Compared with studies using general symptom checklists, the RSI’s structured, scored format captures symptom severity rather than mere presence, providing finer granularity of pharyngolaryngeal impact.

Several mechanisms may explain the pronounced throat symptoms. Omicron generates a higher viral load in the nasopharynx and more prolonged viral shedding than Delta (9). It also induces enhanced neutrophil degranulation, T- and B-cell activation, and upregulation of the interleukin inflammatory cascade, exacerbating pharyngeal and laryngeal mucosal inflammation (10). SARS-CoV-2’s neurotropic properties (11, 12) and associated respiratory microbiota dysbiosis may further compound mucosal vulnerability (13). Additionally, Omicron’s spike protein mutations alter ACE2 receptor binding affinity—highly expressed in oral and nasal tissues—potentially modifying viral tropism at pharyngeal sites (14).

Studies have shown that males are usually more sensitive to severe outcomes at younger and older ages, while females are more susceptible to severe outcomes during their reproductive years (15). Female sex in our study was associated with higher RSI scores during infection (*p* = 0.010) and showed a borderline association in univariate regression (*β* = 1.96, *p* = 0.010), though this did not reach significance in the multivariable model (*p* = 0.058). Psychological factors, including anxiety and depression, may modulate sympathetic nervous activity and throat symptom perception (16), though no difference was found between healthcare workers and non-healthcare workers.

As shown in Table 2, throat symptom duration was prolonged in patients with RSI > 13 (≈50% > 15 days) and positively correlated with RSI scores, whereas prior COVID-19 infection frequency did not influence RSI scores. Post-Omicron symptom profiles in US (17) and Norwegian (18) cohorts largely overlapped, predominantly featuring cough, fatigue, nasal congestion, fever, dyspnea, and anosmia/ageusia, alongside gastrointestinal and musculoskeletal manifestations. The associations of fatigue, nasal congestion, and muscle pains with greater RSI increase reflect the intensity of the systemic inflammatory response. Nasal congestion (stuffy/runny nose) was the strongest independent factor associated with RSI increase (*β* = 3.30). This is mechanistically plausible: nasal obstruction impairs mucociliary clearance and promotes postnasal drip, directly irritating the pharyngeal mucosa and contributing to throat clearing, excess mucus, and cough—all captured by RSI items 2, 3, and 7. Similar associations between upper airway congestion and pharyngolaryngeal symptoms have been reported in allergic rhinitis and post-viral rhinosinusitis, supporting the biological plausibility of this association. Fatigue and muscle pain were independently associated with ΔRSI. These likely reflect overall disease severity; patients with more severe systemic illness also experience more severe pharyngolaryngeal involvement (19). We refrain from speculating about the specific mechanistic pathway linking systemic myalgia to pharyngeal symptoms, as this cannot be established from the current observational data. A prior retrospective study reported that RSI > 13 was associated with worse clinical outcomes in hospitalized COVID-19 patients (20), suggesting RSI may serve as a proxy for systemic inflammatory burden. Erosive mucosal changes of the glottis and supraglottis were observed in all 18 patients who underwent laryngoscopy. These findings may reflect severe local inflammatory injury during SARS-CoV-2 infection; however, in the absence of a control group, histological confirmation, or lesion-specific viral testing, causal attribution to direct cytopathic effect or Omicron-specific injury cannot be established. These observations are reported as exploratory and require confirmation in future controlled studies.

Omicron infection generally causes milder disease than Delta, suggesting progressive attenuation of SARS-CoV-2 pathogenicity. However, its extensive mutational burden—particularly 30 spike protein substitutions—enhances transmissibility and immune evasion (21), driving case surges and complicating epidemic control. Despite this immune escape, vaccination retains partial efficacy (22). Notably, one-dose vaccination was associated with a greater increase in RSI score (*β* = 4.96, 95% CI 0.99–8.92, *p* = 0.015), whereas ≥2 doses showed no significant association. This observation should be interpreted with considerable caution. The single-dose subgroup was small (*n* = 10), and the wide confidence interval (0.99–8.92) reflects this uncertainty. Vaccination timing was not recorded, and vaccine type, time since vaccination, prior infection history, and health-seeking behavior may confound this association. Because each dose category was tested as an independent binary comparison rather than within a single ordered model, these results should not be interpreted as evidence of a dose-dependent relationship. Moreover, because five separate dose-category comparisons were performed without a joint reference model, multiplicity must be considered: applying a Bonferroni-corrected threshold (*p* < 0.01) for five comparisons, the one-dose association (*p* = 0.015) would no longer be statistically significant. We therefore present this finding strictly as an exploratory sensitivity analysis rather than a confirmed association. We do not claim that single-dose vaccination increases throat symptom risk, nor do we assert that booster vaccination is protective.

Several limitations warrant mention. First, although 24-h dual-probe PH is currently the gold standard for detecting LPR, because it is expensive and invasive, we used it only in participants with RSI > 13 and/or RFS > 7; participants with lower scores were classified as LPR-screened-negative, which may have missed a small proportion with subclinical LPR. Readers should therefore note that our cohort is more accurately described as “screened negative for LPR” rather than “confirmed LPR-free.” However, there are insufficient studies to show a correlation between the incidence of LPR and COVID-19 infections (23). Second, with regard to the literature reporting that patients with RSI > 13 have a poorer prognosis after SARS-CoV-2 infection (20), we were unable to argue against the literature’s viewpoint, as the severity of the patients’ condition was not documented in our study. Third, although the vaccination rate of COVID-19 is high in China, we did not record the time of vaccination, which has an impact on the study of the correlation between the vaccine and throat symptoms. Fourth, the online self-administration of the RSI via WeChat may introduce self-report bias, as participants might interpret or recall symptoms differently without direct clinical assessment. Fifth, the follow-up RSI was completed ≥4 weeks after infection onset, requiring participants to retrospectively recall acute-phase symptoms; recall bias may have affected symptom reporting. Sixth, if baseline throat symptom levels changed independently between the baseline assessment and infection onset (e.g., due to aging, lifestyle changes, or interim illnesses), the before-after comparison may not solely reflect the effect of SARS-CoV-2 infection. Finally, the homogenous Chinese sample limits the extrapolation of our results; future studies should include more racially and ethnically diverse populations and validate findings across different variants and healthcare settings to enhance generalizability.

## 5 Conclusion

Although the acute phase of the COVID-19 pandemic has subsided, systematic documentation of associated throat symptoms remains clinically pertinent for informing management of potential long-term otorhinolaryngological sequelae and for providing a rigorous empirical record for future academic and clinical reference. This study demonstrates that the RSI—a structured symptom-burden instrument validated for LPR assessment—provides a clinically meaningful framework for quantifying throat symptom burden associated with SARS-CoV-2 infection, while recognizing that it has not been validated as a COVID-19 outcome measure.

Our findings show that SARS-CoV-2 infection during the Omicron-dominant period significantly increases RSI scores in LPR-screened-negative patients, with troublesome cough, throat clearing, and excess throat mucus emerging as the dominant symptoms. Clinicians should be alert to patients presenting with concurrent fatigue, nasal congestion, and muscle pain during SARS-CoV-2 infection, as these features were independently associated with greater throat symptom burden and may identify individuals who could benefit from early, targeted supportive management.

## Data Availability

The original contributions presented in the study are included in the article/Supplementary material, further inquiries can be directed to the corresponding author.
